# Primary Renal Fibrosarcoma with Massive Tumor Size and Inferior Vena Cava Extension Mimicking Renal Cell Carcinoma: A Rare Case Report

**DOI:** 10.15586/jkc.v13i1.430

**Published:** 2026-01-12

**Authors:** Kapil Rathore, Ketan Mehra, Manoj Yadav, Devashish Kaushal, Deepti Joshi, Kumar Madhavan, Nikita Shrivastava, Chandana

**Affiliations:** 1 Department of Urology, All India Institute of Medical Sciences (AIIMS), Bhopal, India;; 2 Department of Pathology, All India Institute of Medical Sciences (AIIMS), Bhopal, India

**Keywords:** fibrosarcoma, inferior vena cava thrombus, radical nephrectomy, renal neoplasm, spindle cell tumor

## Abstract

Primary renal fibrosarcoma is an exceedingly rare malignant mesenchymal tumor that accounts for only 1–3% of adult renal malignancies, often mistaken for sarcomatoid renal cell carcinoma (RCC) or leiomyosarcoma due to overlapping morphology. Thus, accurate disease diagnosis is crucial for its management. We report a case of a 49-year-old female, a chronic smoker, who presented with right flank pain and progressive abdominal swelling. Clinical examination revealed a firm mass in the right abdomen, and contrast-enhanced computed tomography demonstrated a large exophytic right renal mass with areas of necrosis, infiltration of the psoas muscle, and extension of tumor thrombus into the inferior vena cava (IVC). The patient underwent right radical nephrectomy with IVC thrombectomy, and a 20 × 15 cm irregular renal mass with 2 cm IVC thrombus was excised intraoperatively. Histopathology revealed a high-grade spindle cell neoplasm with interlacing fascicles and herringbone patterns, brisk mitoses (10–12 mitotic figures per 10HPF), and 20% necrosis. Immunohistochemistry showed diffuse vimentin positivity with negative staining for epithelial, myogenic, neural, and renal lineage markers, confirming the diagnosis of high-grade primary renal fibrosarcoma. This case is notable for being the largest renal fibrosarcoma reported to date, with rare IVC extension, features typically associated with advanced RCC rather than fibrosarcoma. Despite aggressive pathology, no metastases were identified, and the patient remained recurrence-free at 6 months postoperatively, with chemotherapy reserved for recurrence or metastasis. This report emphasizes the diagnostic challenges, surgical complexity, and clinical significance of primary renal fibrosarcoma and highlights the importance of including it in the differential diagnosis of large renal masses with vascular involvement.

## Introduction

Renal fibrosarcoma is a rare malignant mesenchymal tumor arising from the fibrous connective tissue of the kidney capsule. Primary sarcomas of the kidney constitute only 1–3% of adult renal malignancies, with leiomyosarcoma being the most common histological type and fibrosarcoma being a very rare tumor (1, 2).

Due to overlapping morphological features, primary renal fibrosarcoma must be carefully differentiated from sarcomatoid renal cell carcinoma (RCC) and leiomyosarcoma. This distinction has significant prognostic and therapeutic implications. Immunohistochemistry plays a crucial role in this differentiation, as fibrosarcoma typically expresses vimentin but not cytokeratins or smooth muscle markers. We report a rare case of a 49-year-old female with a large right renal mass and inferior vena cava (IVC) thrombus, initially presumed to be RCC.

## Case Report

A 49-year-old female, chronic smoker for the past 20 years with no significant medical history, presented with a complaint of right flank pain persistent for 3 months. The pain was intermittent in nature and temporarily relieved with medication. One month before the presentation, she noticed a gradually increasing abdominal swelling. There was no associated history of hematuria, fever, or weight loss.

### Clinical findings

On clinical examination, the patient was stable and afebrile. Abdominal palpation revealed a firm, nontender mass measuring approximately 15 × 10 cm in the right hypochondriac and lumbar region.

### Diagnostic assessment

Laboratory investigations revealed normal renal function with serum creatinine levels of 0.79 and 0.87 mg/dL on separate occasions. Hemoglobin was approximately 12.8 g/dL, with normal white blood cell and platelet counts.

**Figure 1:**
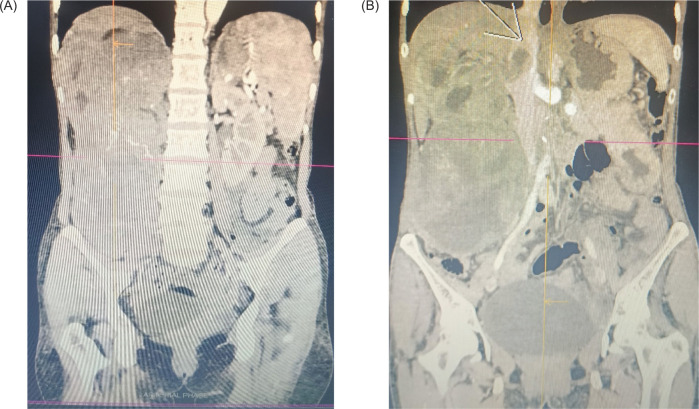
CECT abdomen showing (A) Large approx. 13.4 x 10.8 x 19 cm (APxTRxCC) exophytic heterogeneously enhancing soft tissue lesion noted involving the right kidney, and (B) Tumor thrombus is contiguously extending into the right renal vein and superiorly into the IVC for a length of 2.3 cm.

A contrast-enhanced computed tomography (CECT) scan of the thorax and abdomen revealed a large lobulated, heterogeneously enhancing exophytic mass arising from the right kidney, measuring 13.4 × 10.8 × 19 cm (Figure 1). The lesion showed non-enhancing necrotic areas and was noted to infiltrate the adjacent right psoas muscle. Importantly, a tumor thrombus was also observed extending superiorly 2.3 cm into the IVC. There was no radiological evidence of hepatic or nodal metastases.

**Figure 2:**
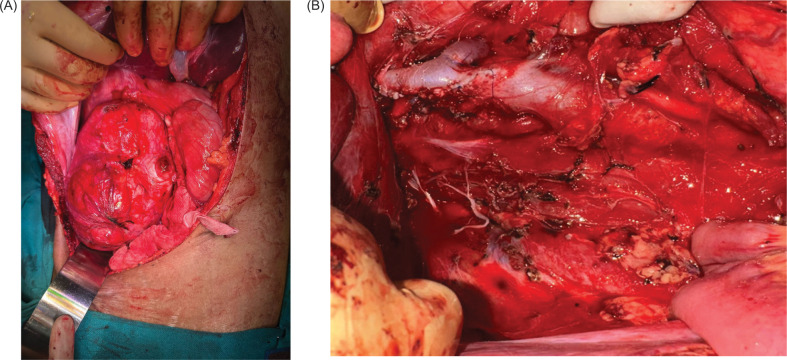
Intraoperative image showing (A) a large right renal mass, and (B) the renal bed post specimen removal and sutured IVC defect.

The patient underwent a right open radical nephrectomy with IVC thrombectomy. Intraoperatively, a large, irregular, nodular mass measuring 20 × 15 cm was found arising from the mid and lower pole of the right kidney (Figure 2). A single right renal artery and double right renal veins were identified. A 2 cm thrombus was noted within the IVC, which was successfully removed during the procedure.

### Histopathology

**Figure 3:**
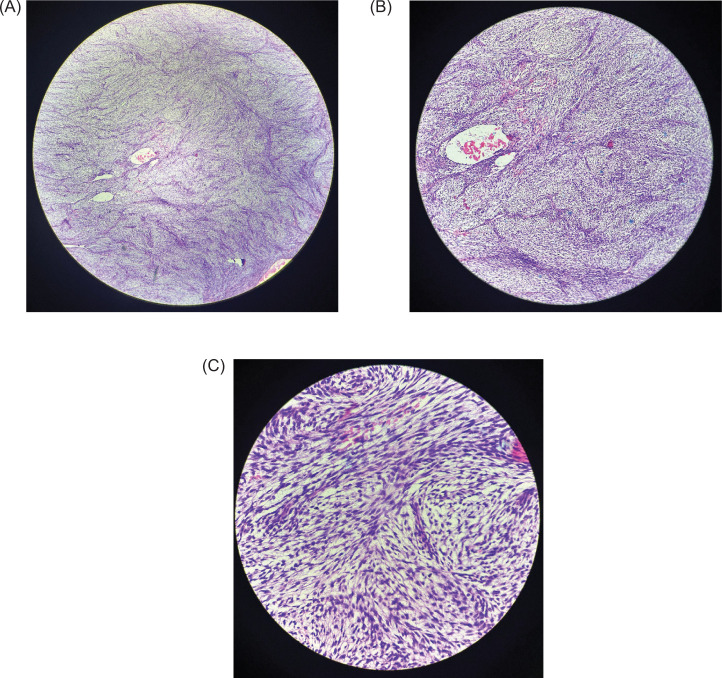
(A) 4x magnification (B) 10x magnification (C) 40x magnification microscopic view of renal parenchyma entirely replaced by an infiltrative tumor arranged in interlacing fascicles and bundles, also in a herringbone pattern, separated by dense collagenised (keloid-like) and hyalinized stroma. The tumor cells are spindled in shape with elongated nuclei, moderate pleomorphism with moderate to scant amount of cytoplasm. Mitotic activity is brisk (10–12 mitotic figures per 10 high-power fields, consistent with high-grade sarcoma). Areas of coagulative necrosis are seen.

Gross pathological examination of the nephrectomy specimen, measuring 24 × 15 × 10 cm, revealed a bosselated, solid cystic tumor replacing the entire kidney. Areas of hemorrhage and necrosis were evident, although the ureter and perinephric fat were uninvolved grossly. Microscopically, the tumor was composed of spindle cells arranged in interlacing fascicles and herringbone patterns (Figure 3). The tumor cells displayed elongated, hyperchromatic nuclei with scant cytoplasm and moderate nuclear pleomorphism. Brisk mitotic activity and approximately 20% tumor necrosis were observed. The tumor demonstrated a mitotic rate of 10–12 mitotic figures per 10 high-power fields (HPF), consistent with high-grade sarcoma. The tumor noted in the renal vein, but the perinephric fat and the single retrieved lymph node were free of tumor. The ipsilateral adrenal gland was uninvolved.

**Figure 4:**
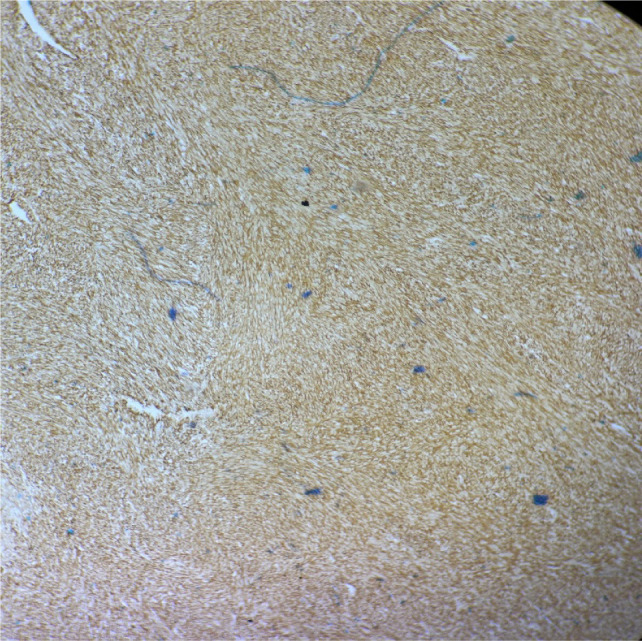
Microscopic 10x magnification of IHC vimentin positivity.

Immunohistochemical analysis supported a mesenchymal origin of the tumor. The tumor cells showed diffuse positivity for vimentin, while markers for epithelial (CK7, EMA), myogenic (desmin, SMA), neural (S100), and other renal lineage markers (PAX8, CD10) were negative. Additional markers, including CD117, TLE1, and ER were also negative (Figure 4).

Based on the morphological features and immunoprofile, a final diagnosis of high-grade primary renal fibrosarcoma with renal vein invasion was made. Given the rarity of primary fibrosarcoma of the kidney and its aggressive behavior, close follow-up was advised. At 6 months follow-up, the patient was recurrence-free and doing well, and was kept on postoperative surveillance with CECT scan of the abdomen, pelvis, and thorax after discussion in the multidisciplinary tumor board.

## Discussion

Primary renal fibrosarcoma is an exceptionally rare malignant mesenchymal tumor, comprising only 1–3% of adult renal malignancies, with leiomyosarcoma being more common (1, 2). Historically, many such tumors were misclassified as “renal sarcoma,” and in some cases, as sarcomatoid RCC. With the advent of immunohistochemistry (IHC), it is now possible to reliably differentiate these entities. Fibrosarcoma typically shows diffuse vimentin positivity and is negative for epithelial markers (cytokeratin, EMA) and myogenic markers (desmin, SMA) (1–3).

The present case is remarkable for several reasons:

1. **Massive tumor size** – Measuring 24 × 15 × 10 cm, this is the largest primary renal fibrosarcoma documented in the literature to date, exceeding the more typical range of 10–17 cm reported in most series.
2. **IVC tumor thrombus** – Extension into the IVC is exceptionally rare and, to our knowledge, has not been reported in previously available renal fibrosarcoma case reports.
3. **Relatively young age** – Most cases occur in patients over 50 years, whereas our patient was only 49 years old.
4. **Absence of distant metastasis** – Despite large size, high-grade histology, and vascular invasion, no metastases were identified, which is unusual given the aggressive biology of this tumor.

Clinically, symptoms are nonspecific, most often flank pain, abdominal mass, or hematuria (1–3). Imaging findings often mimic RCC, and histopathology with IHC remains the gold standard for diagnosis (3). Radical nephrectomy is the mainstay of treatment; adjuvant chemotherapy or radiotherapy has not demonstrated a clear survival benefit, and prognosis remains poor, with 5-year survival rates <10% (1, 2).

Although adjuvant chemotherapy is often discussed for high-grade soft-tissue sarcomas, including renal fibrosarcoma, there is no tumor-specific regimen due to its rarity. Available evidence extrapolates from soft-tissue sarcoma protocols, typically using doxorubicin–ifosfamide combinations or gemcitabine–docetaxel, but with inconsistent outcomes. In this case, after reviewing the pathology and complete surgical resection, the Institutional Multidisciplinary Tumor Board determined that there was insufficient evidence to justify immediate chemotherapy. Therefore, the patient was placed on postoperative surveillance, with systemic therapy reserved only if recurrence or metastasis develops (2, 4–7).

A comparison of our case with previously reported primary renal fibrosarcoma cases is shown in Table 1.

**Table 1:** Comparison of present case with reported primary renal fibrosarcoma cases.

References	Year	Age	Gender	Size (cm)	Symptoms	Treatment	Pathologic diagnosis	Side	Metastatic	Follow up (months)
Chaudhari et al. (1)	2013	70	M	17 × 10 × 6	Abdominal swelling and pain	Radical nephrectomy	Vim (+), CK (−), desmin (−)	Right	-	-
Agarwal et al. (2)	2008	54	M	17.5 × 12.5 × 9	Intermittent hematuria and pain in the lumbar region	Radical nephrectomy	Vim (+), CK (−), desmin (−), EMA (−), SMA (−), PAN (−), S100 (−)	Right	-	6
Jiang et al. (3)	2021	72	M	3.5 × 2.5 × 2	Severe hydronephrosis	Radical nephro-ureterectomy	CK5/6 (+), CK (+), CK8/18 (+), CK 7 (+), Vimentin (+++), Ki-67 (85%+), Desmin (+), HMB-45 (−)	Right	-	3
Gupta et al. (8)	2013	75	F	15 × 10	Vague abdominal discomfort	Radical nephrectomy	Vim (+), Ki-67 (+), CK (−), Desmin (−), HMB-45 (−), SMA (−)	Right	-	-
Ares Valdés et al. (9)	2003	53	F	10	Left flank pain, fever and palpable mass	Radical nephrectomy	Not specified	Left	Brain	Died after 24 months
Kaneoya et al. (10)	1986	64	F	Not specified	Hematuria	Nephrectomy	Not specified	Left	Peritoneal	Died after 4 months
The present case	2025	49	F	24 × 15 × 10 with IVC extension	Right flank pain and palpable lump	Radical nephrectomy with IVC thrombectomy	Vim (+), SMA (−), PAX8 (−), EMA (−), TLE1 (−), S100 (−), CD117 (−), ER (−), CD10 (−), CK7 (−)	Right	-	6 months

From this comparison, the present case clearly stands out due to the largest tumor size reported and the rare IVC extension in primary renal fibrosarcoma, features that significantly complicate surgical management and are more typical of advanced RCC than fibrosarcoma. These findings underscore the need for a high index of suspicion and meticulous surgical planning when encountering large renal masses with vascular involvement.

## Conclusion

Primary renal fibrosarcoma is a rare, aggressive, malignant tumor that poses significant diagnostic and therapeutic challenges. Differentiation from sarcomatoid RCC and other spindle cell neoplasms requires a comprehensive immunohistochemical panel, as imaging alone is often inconclusive. Successful radical nephrectomy with IVC thrombectomy, in select cases, remains the primary management in primary renal fibrosarcoma. This case contributes valuable evidence to the limited literature and highlights the importance of considering primary renal fibrosarcoma in the differential diagnosis of large renal masses with vascular extension.
